# Acute Hyperglycemia-Induced Inflammation in MIO-M1 Cells: The Role of Aldose Reductase

**DOI:** 10.3390/ijms26146741

**Published:** 2025-07-14

**Authors:** Francesca Felice, Gemma Sardelli, Francesco Balestri, Lucia Piazza, Mario Cappiello, Rossella Mosca, Antonella Del Corso, Martina Avanatti, Simone Allegrini, Roberta Moschini

**Affiliations:** 1 Biochemistry Unit, Department of Biology, University of Pisa, 56123 Pisa, Italy; francesca.felice@unipi.it (F.F.); francesco.balestri@unipi.it (F.B.); luciapiazza@cnr.it (L.P.); mario.cappiello@unipi.it (M.C.); r.mosca@studenti.unipi.it (R.M.); antonella.delcorso@unipi.it (A.D.C.); m.avanatti@studenti.unipi.it (M.A.); roberta.moschini@unipi.it (R.M.); 2 Interdepartmental Research Center Nutrafood “Nutraceuticals and Food for Health”, University of Pisa, 56124 Pisa, Italy; 3 Center for Instrument Sharing University of Pisa (CISUP), Lungarno Pacinotti, 43/44, 56126 Pisa, Italy; 4 National Research Council—CNR, Institute on Clinical Physiology, via Moruzzi 1, 56124 Pisa, Italy; gemmasardelli@cnr.it

**Keywords:** AKR1B1, macroglia, MIO-M1 cells, inflammation

## Abstract

Diabetic retinopathy (DR), traditionally considered a microvascular complication, is now recognized as a neuroinflammatory disorder involving retinal glial cells. Aldose reductase (AKR1B1), a key enzyme in the polyol pathway, has been implicated in the hyperglycemia-induced inflammatory response in various cell types, although its role in retinal Müller glial cells under acute glucose stress remains unclear. This study investigates AKR1B1 activity and its contribution to inflammatory signaling in MIO-M1 human Müller cells exposed to acute hyperglycemia. AKR1B1 expression and activity, as well as NF-κB activation and COX-2 expression, were evaluated. Sorbinil, a specific AKR1B1 inhibitor, was used to determine the enzyme’s contribution to acute hyperglycemia-induced inflammation. Acute high-glucose treatment significantly increased AKR1B1 activity and sorbitol accumulation without affecting cell viability. In addition, activation of NF-κB and increased expression of cyclooxygenase-2 (COX-2) were observed, both of which were significantly reduced by Sorbinil. Our findings highlight the role of macroglia as active contributors to early inflammatory events in DR and suggest that transient hyperglycemic spikes are sufficient to trigger AKR1B1-dependent glial activation.

## 1. Introduction

Diabetes mellitus is a metabolic disease characterized by elevated blood glucose levels and dysregulation in the metabolism of carbohydrates, proteins, and lipids, leading to impaired homeostasis across multiple metabolic pathways [1]. Among the secondary complications of diabetes, the most prominent, resulting from microvascular damage to small arterial vessels, are retinopathy, nephropathy, peripheral neuropathy, and cataract. Diabetic retinopathy (DR) has a substantial impact on the quality of life of individuals with diabetes and remains the leading cause of vision loss among working-age adults in industrialized nations [2,3]. DR is now recognized as a neurovascular disorder wherein neurodegenerative processes play a pivotal role [4]. Emerging evidence indicates that retinal glial cells, including macroglia (Müller cells and astrocytes) and microglia, undergo significant alterations in response to hyperglycemic conditions [5,6]. Among the macroglial cell types in the retina, Müller glial cells (MGCs) are the most abundant and widely distributed. MGCs span the entire retinal thickness and play a pivotal role in maintaining retinal homeostasis through several mechanisms [6]. The glial responses not only precede overt vascular alterations but also contribute to the progression of DR by disrupting the neurovascular unit’s integrity [7]. In Müller cells, the presence of aldose reductase (AKR1B1) expression, particularly in areas adjacent to the inner limiting membrane, was observed [8]. AKR1B1, the first and rate-limiting enzyme of the polyol pathway, catalyzes the reduction of glucose to sorbitol, which is subsequently converted into fructose by sorbitol dehydrogenase (SORD). Under normoglycemic conditions, this pathway accounts for a minor fraction of glucose metabolism. However, in hyperglycemic states, increased flux through the polyol pathway leads to sorbitol accumulation, osmotic stress, and oxidative damage [9]. AKR1B1 is not only involved in osmoregulation; it also contributes to inflammatory pathways by promoting the production of reactive oxygen species (ROS) and activation of pro-inflammatory transcription factors such as nuclear factor kappa-light-chain-enhancer of activated B cells (NF-κB) [10].

Previous studies have demonstrated that AKR1B1 contributes to inflammation in various ocular tissues. For instance, AKR1B1 inhibition has been shown to attenuate lipopolysaccharide-induced inflammatory responses in retinal microglia [11]. Moreover, in diabetic models, AKR1B1 activity correlates with increased expression of inflammatory cytokines and vascular endothelial growth factor (VEGF), further implicating its role in DR pathogenesis [12]. Interestingly, the role of AKR1B1 in inflammation appears to be cell-type specific. In human lens epithelial cells, AKR1B1 activity did not significantly induce inflammatory markers, suggesting differential regulatory mechanisms across ocular cell types [13]. The potential role of AKR1B1 expressed in retinal MGCs as a mediator of inflammatory responses in the diabetic retina has been suggested. However, a deeper investigation into the involvement of the polyol pathway in macroglial cells exposed to acute glycaemic stress is still lacking in the current literature. The acute hyperglycemic events, although temporary, have been associated with inflammatory activation, oxidative stress, and vascular dysfunction in the diabetic retina [14].

In this study, we aim to elucidate the role of enzymes involved in the polyol pathway, particularly AKR1B1, in mediating acute hyperglycemia-induced inflammation in MIO-M1 cells. Acute hyperglycemia in vitro mimics several in vivo conditions typically observed in diabetic individuals or patients under metabolic stress [15]. To this end, we will explore, by specific biochemical assays, how short-term glycemic stress modulates the polyol pathway and activates downstream inflammatory signaling in macroglia cells, including the use of a specific aldose reductase inhibitor. Through elucidation of the biochemical mechanisms underlying acute hyperglycemic stress, it will be possible to identify potential molecular targets for therapeutic interventions to reduce retinal inflammation in the early stages of DR.

## 2. Results

### 2.1. Effect of Acute Hyperglycemic Stress on Cell Viability

**Figure 1:**
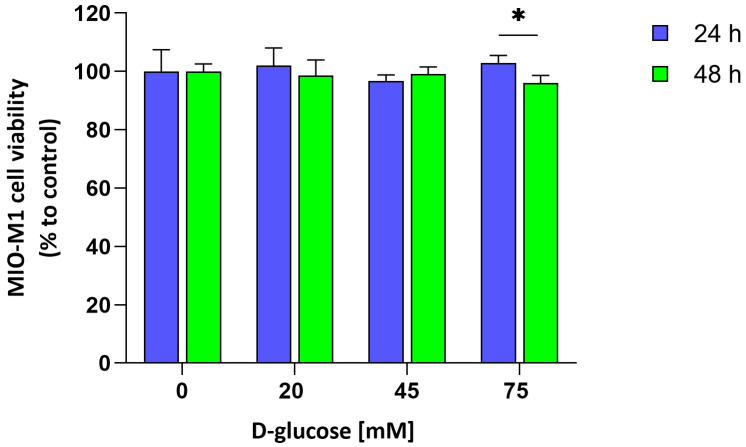
Dose- and time-dependent cell metabolic activity. MIO-M1cells were cultured for 24 h (blue bar) and 48 h (green bar) in the presence of increasing added concentrations of D-glucose (20, 45, and 75 mM). Cell metabolic activity was determined by MTT colorimetric assay and expressed as the percentage of cell viability calculated with respect to control (untreated cells). Data are represented as mean ± SEM of five independent experiments. Statistical analysis was performed through two-way ANOVA, followed by Bonferroni’s post hoc test (* *p* ≤ 0.05).

The assessment of MIO-M1 cells’ tolerance to different glucose concentrations over time was measured by MTT colorimetric assay. Figure 1 shows the viability of MIO-M1 cells exposed to increasing concentrations of D-glucose (20–45–75 mM) for different times (24 and 48 h). Results indicated no significant differences in cell viability between glucose concentrations at each time point, indicating that increasing glucose levels did not induce a concentration-dependent cytotoxic effect at 24 or 48 h. However, a significant reduction in viability was observed at 75 mM between 24 and 48 h (* *p* < 0.05). For this reason, a 24 h exposure was chosen to investigate early cellular responses to acute hyperglycemic stress.

### 2.2. Effect of Acute Hyperglycemia on AKR1B1 and SORD Expression in MIO-M1 Cells

Enzymatic activity assays performed under basal conditions revealed measurable specific activities in MIO-M1 cell extracts for AKR1B1 and SORD (23.4 ± 2.2 mU/mg and 18.8 ± 1.8 mU/mg, respectively) using L-idose and fructose as substrates, with L-idose employed as a surrogate substrate for AKR1B1 due to its higher reaction rate compared to glucose in in vitro assays.

The minimal effective glucose concentration was determined by assessing NF-κB activation across a range of glucose levels (0 to 75 mM). As reported in Figure S1, 75 mM glucose was identified as the lowest concentration capable of significantly activating NF-κB and was therefore selected to evaluate its effect on AKR1B1 expression and activity.

**Figure 2:**
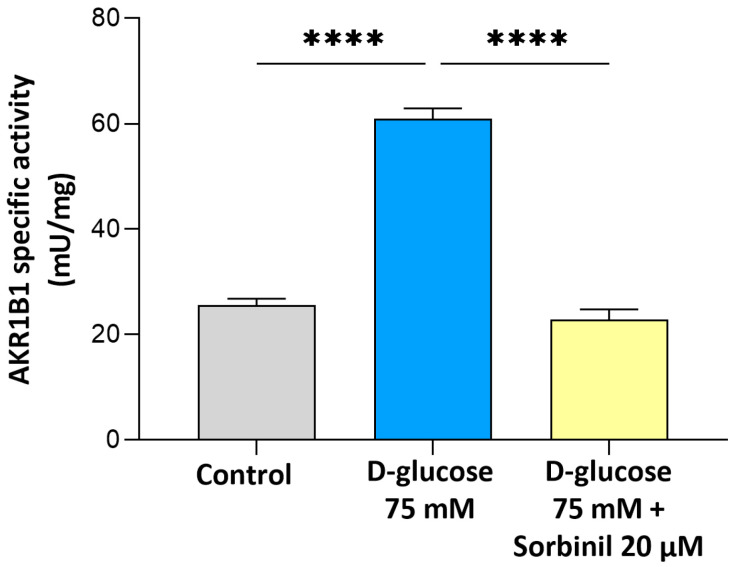
Specific activity of AKR1B1. MIO-M1 cells were incubated under basal conditions (control) or exposed to 75 mM D-glucose, with or without a 24 h pre-treatment with 20 µM Sorbinil or 0.05% DMSO (vehicle control). After 24 h, cells were harvested, and AKR1B1-specific activity was measured as detailed in Materials and Methods. Enzymatic activity is expressed as mU normalized to mg of protein and reported as the mean ± SEM of three independent experiments. Statistical analysis was performed through one-way ANOVA followed by Dunnett’s post hoc test (**** *p* ≤ 0.0001 vs. D-glucose 75 mM).

As reported in Figure 2, AKR1B1enzymatic activity was significantly increased in response to acute hyperglycemia (75 mM D-glucose, for 24 h) compared to cells grown under normal glucose condition (5 mM D-glucose and PBS as vehicle) (60.9 ± 3.4 mU/mg vs. 25.6 ± 2.1 mU/mg; *p* ≤ 0.0001). This increase in enzymatic activity can be ascribed to an upregulation of AKR1B1 protein expression, as confirmed by Western blot analysis. Indeed, densitometric quantification revealed a higher AKR1B1 expression in cells exposed to high glucose (about a two-fold increase), as shown in the Supplementary Materials (Figure S2). This increase in activity was significantly reduced in cells pre-treated with the AKR1B1 inhibitor Sorbinil (20 μM in DMSO) for 24 h. As shown in Figure 2, Sorbinil effectively restored AKR1B1 activity to levels comparable to the normoglycemic control.

**Figure 3:**
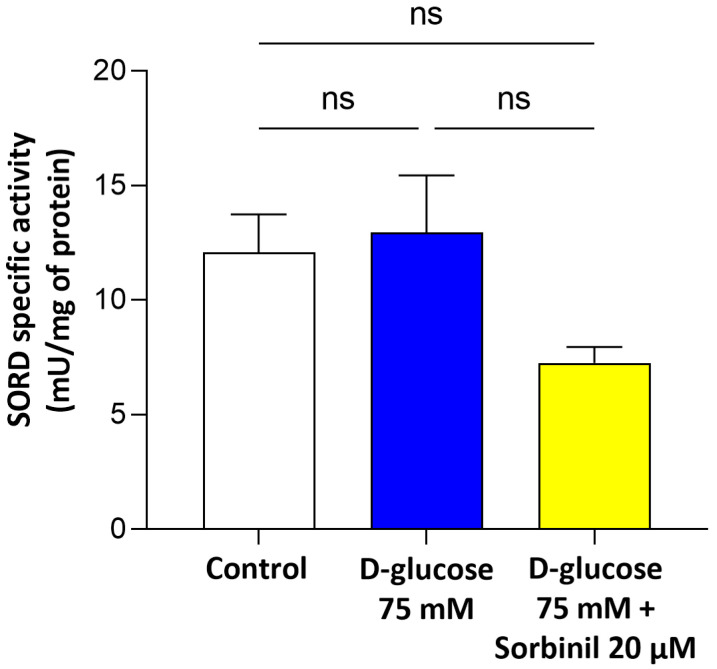
Specific activity of SORD. MIO-M1 cells were incubated under basal conditions (control) or exposed to 75 mM D-glucose, with or without a 24 h pre-treatment with 20 µM Sorbinil or 0.05% DMSO (vehicle control). After 24 h, cells were harvested, and SORD-specific activity was measured. Enzymatic activity was measured as reported in Materials and Methods and expressed as mU normalized to mg of protein. Data are reported as mean ± SEM of at least three independent experiments. Statistical analysis was performed through one-way ANOVA followed by Dunnett’s post hoc test (ns: not significant).

Figure 3 shows SORD enzymatic activity. No statistically significant difference (ns) was observed among all conditions, suggesting that high glucose (75 mM for 24 h) and Sorbinil (20 µM) do not alter SORD activity.

**Figure 4:**
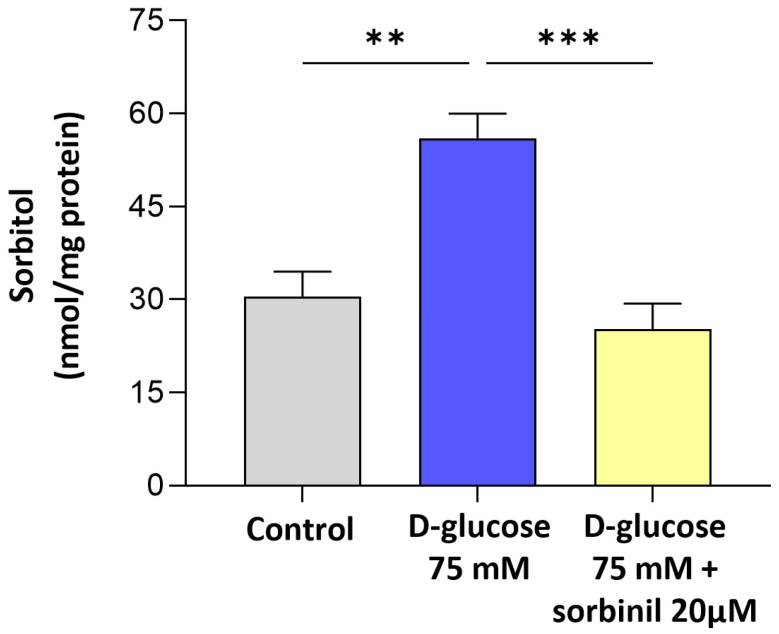
Sorbitol levels in MIO-M1 cells. MIO-M1 cells were incubated under basal conditions (control) or exposed to 75 mM D-glucose, with or without a 24 h pre-treatment with 20 µM Sorbinil or 0.05% DMSO (vehicle control). After 24 h, cells were harvested, and sorbitol content was measured. Sorbitol content is expressed as nmoles of sorbitol normalized to mg of proteins and reported as mean ± SEM of at least six independent experiments. Statistical analysis was performed through one-way ANOVA followed by Dunnett’s post hoc test (** *p* ≤ 0.01 and *** *p* ≤ 0.001 vs. D-glucose 75 mM).

The effect of acute hyperglycemia on AKR1B1 activity was confirmed by measuring the intracellular sorbitol accumulation in MIO-M1 cells' crude extracts. MGCs were incubated with 75 mM D-glucose for 24 h, with or without a 24 h pre-treatment with 20 µM Sorbinil or DMSO 0.05% as vehicle. As reported in Figure 4, intracellular sorbitol accumulation in MIO-M1 cells was significantly higher compared to control cells (*p* ≤ 0.005). Pre-treatment with 20 μM Sorbinil completely abolishes sorbitol accumulation induced by acute hyperglycemia (*p* ≤ 0.001 vs. D-glucose).

### 2.3. Role of AKR1B1 on NF-κB Activation in Acute Hyperglycemic Conditions

**Figure 5:**
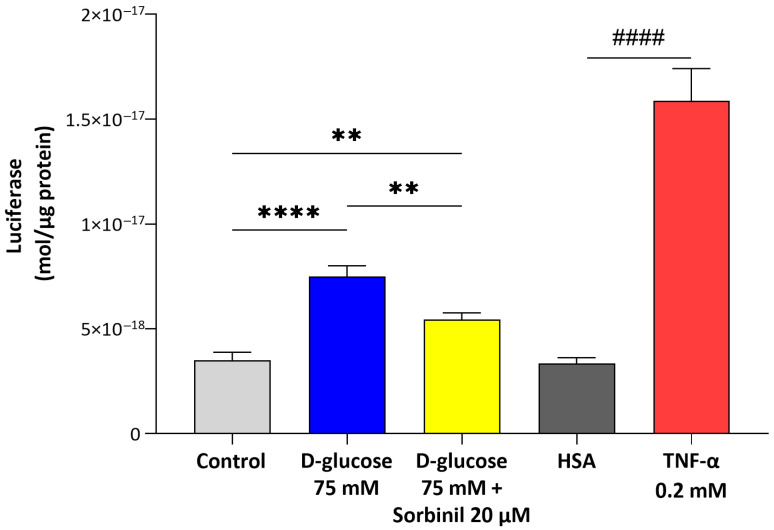
NF-κB activation in acute hyperglycemic conditions. MIO-M1 cells were incubated with 75 mM D-glucose (or PBS) after a 24 h pre-incubation with 20 µM Sorbinil or DMSO 0.05% and with 0.2 mM TNF-α (or HSA 0.001%) for 24 h. Moles of luciferase normalized to µg of proteins are expressed as mean ± SEM of measures of at least six independent experiments. Statistical analysis was performed through one-way ANOVA followed by Tukey’s post hoc test (** *p* ≤ 0.01, **** *p* ≤ 0.0001) or Student’ *t*-test (#### *p* ≤ 0.0001).

To evaluate the role of AKR1B1 in mediating acute hyperglycemia-induced inflammation, NF-κB activation was evaluated in MIO-M1 cells stably transfected with a luciferase reporter construct. The inflammatory response was also supported by NF-κB activation upon TNF-α stimulation. Cells treated with 75 mM D-glucose for 24 h displayed a significant increase in NF-κB-dipendent luciferase activity compared to controls (untreated cells). This activation was significantly reduced in the presence of Sorbinil (20 μM), as reported in Figure 5.

**Figure 6:**
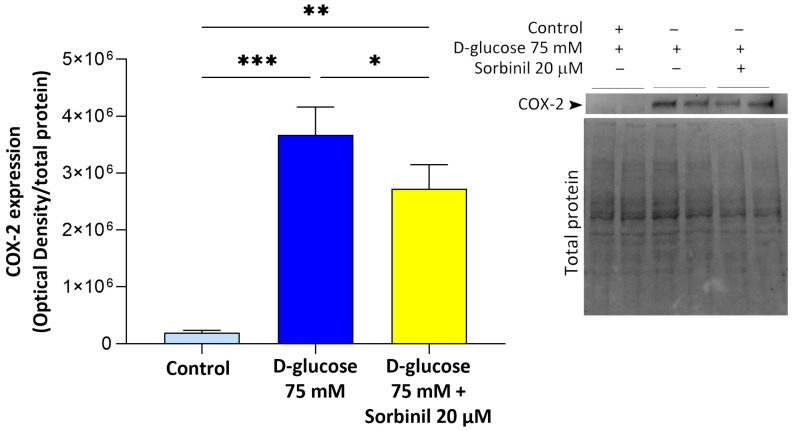
Effect of Sorbinil on COX-2 protein expression. MIO-M1 cells were incubated alone or with 75 mM D-glucose and pre-treated with 20 µM Sorbinil or DMSO 0.05% for 24 h. After 24 h, cells were harvested, and COX-2 protein level was determined through Western blot analysis. Densitometric analysis of COX-2 expression, normalized to total protein, was reported as optical density (OD) of AKR1B1 band expression. Data are reported as mean ± SEM of at least three independent experiments in duplicate. Statistical analysis was performed through one-way ANOVA followed by Tukey’s post hoc test (* *p* ≤ 0.05; ** *p* ≤ 0.01; *** *p* ≤ 0.001).

Furthermore, to assess whether Sorbinil exerts an inhibitory effect on AKR1B1-induced inflammation, COX-2 expression—an additional marker of inflammation and direct target of NF-κB activation—was assessed by Western blot analysis. As shown in Figure 6, acute hyperglycemic conditions induced a significant increase in COX-2 protein expression (*p* ≤ 0.001), which was significantly reduced by 20 µM Sorbinil pre-treatment (*p* ≤ 0.05).

## 3. Discussion

In this study, the effect of acute hyperglycemia on the onset of the inflammatory response in human Müller glial cell line MIO-M1 has been investigated, highlighting the role of aldose reductase (AKR1B1) as a key mediator of this process. Acute hyperglycemia would not only cause direct damage to the human body but also may develop into chronic diseases such as diabetes. Among the various stressors contributing to neuronal damage in DR, a microvascular complication of diabetes, acute hyperglycemia represents a crucial metabolic insult capable of triggering early pathogenic responses in retinal cells. Glial cells play a central role in the early pathophysiological response to hyperglycemia, revealing metabolic imbalance and coordinating inflammatory signaling [16].

AKR1B1, the first enzyme of the polyol pathway, has been implicated in oxidative stress and inflammation under hyperglycemic conditions [17]. Our findings support the idea that even short-term hyperglycemic exposure is sufficient to activate pro-inflammatory pathways in Müller cells, which may contribute to the early inflammatory milieu of DR [18,19]. These early responses, although initially intended as protective or adaptive, may evolve into detrimental processes that exacerbate retinal neurodegeneration and vascular dysfunction as the disease progresses [20].

Our study provides new evidence that AKR1B1 actively contributes to the inflammatory response of retinal Müller glial cells under acute hyperglycemic conditions. Although the expression of AKR1B1 in Müller cells has been previously reported, its functional role as a mediator of inflammation within these macroglial cells remains insufficiently characterized. Our findings fill this gap by demonstrating that acute glucose increment enhances AKR1B1 activity and promotes inflammatory signaling in human MIO-M1 cells, independent of changes in cell viability or sorbitol dehydrogenase expression.

It is important to highlight that the present study is based on previous evidence indicating that the polyol pathway may modulate glial cell behavior in DR [11]. While chronic hyperglycemia has been widely linked to oxidative stress and inflammation, acute glycemic spikes—often experienced during poor glycemic control—are now recognized as pathologically significant. Transient hyperglycemic episodes have been shown to trigger vascular dysfunction and inflammatory activation in the diabetic retina [14]. In this context, our data reveal that even short-term glucose elevation is sufficient to activate nuclear factor-κB (NF-κB) and cyclooxygenase-2 (COX-2) pathways in Müller cells, suggesting that macroglial responses may represent an early and sensitive component of the retinal inflammatory cascade.

The finding that sorbitol builds up under high-glucose conditions despite unchanged SORD expression indicates a bottleneck in the polyol pathway, where increased AKR1B1 activity drives metabolic imbalance. This is in line with previous reports in other cell types [21], but our data extends this concept specifically to retinal glia. Notably, the contribution of AKR1B1 to inflammatory responses appears to be cell type-dependent: while in retinal glia we observed a clear link between AKR1B1 activity and inflammation, in our previous studies on lens epithelial cells, we found that inflammation was independent of AKR1B1 activity. These findings highlight the importance of considering the specific cellular context when investigating the role of AKR1B1 in glucose-induced stress responses and inflammation. In particular, the pharmacological inhibition of AKR1B1 with Sorbinil not only reduced sorbitol levels but also markedly suppressed NF-κB activation and COX-2 upregulation, directly implicating AKR1B1 in the pro-inflammatory signaling elicited by acute hyperglycemia. These findings indicate that AKR1B1 acts both as a metabolic sensor and an upstream regulator of inflammatory gene expression in Müller cells.

Although numerous studies have focused on chronic changes associated with advanced stages of the disease, our findings highlight the importance of early and transient metabolic alterations and the macroglial response to these changes. In consideration of the important role played by Müller cells in maintaining retinal homeostasis, including the regulation of ion balance, neurotransmitter recycling, and neurovascular coupling, their early activation by AKR1B1-mediated pathways could set the stage for later neurovascular dysfunction.

Importantly, the sensitivity of Müller cells to AKR1B1 inhibition under hyperglycemic stress suggests a window of opportunity for early intervention. Current treatments of DR mainly target advanced stages and vascular complications; however, modulating AKR1B1 activity during acute glycemic fluctuations could potentially mitigate glial-driven inflammation and preserve retinal integrity.

In conclusion, our study reveals a central role of the polyol pathway, and particularly AKR1B1, in contributing to inflammatory signaling in retinal Müller glial cells following acute hyperglycemic exposure. The polyol pathway can represent the mechanism for the changes seen in the Müller cells of human diabetic retinas.

While the use of the MIO-M1 cell line provides a reliable model for initial investigations, we acknowledge its limitations in fully replicating the behavior of primary cells. Future studies in primary human Müller cells and murine models of diabetic retinopathy will be essential to confirm and refine these findings, thereby enhancing their physiological and translational relevance.

By identifying AKR1B1 as a key mediator of both metabolic and pro-inflammatory responses, we highlight a potential early therapeutic target in the management of diabetic retinopathy. These results emphasize the importance of viewing glial cells not merely as bystanders, but as key contributors to the early development of retinal pathology in diabetes.

## 4. Materials and Methods

### 4.1. MIO-M1 Cell Culture and Transfection

Cell culture media, fetal bovine serum (FBS), penicillin/streptomycin solution, gentamicin, and glutamine were purchased from Euroclone (Pero, Italy), hygromycin was from Merck Life Science (Milan, Italy). In vitro studies were performed using spontaneously immortalized human Müller cells (MIO-M1), kindly provided by Prof. Massimo Dal Monte (Department of Biology, General Physiology Unit, University of Pisa, Pisa, Italy) [22].

Stable transfection of MIO-M1 cells was performed as previously reported for human lens epithelial cells [13]. Briefly, MIO-M1 cell lines were transfected with a pGL4.32[luc2P/NF-κB-RE/Hygro] plasmid (Promega, Madison, WI, USA), which contains a hygromycin and ampicillin resistance gene. This plasmid contained five copies of the NF-κB response element (NF-κB-RE), enhancing transcription of the luc2P reporter gene encoding *Photinus pyralis* luciferase. To confirm that the luminescent signal originated specifically from the expression of the luc2P reporter gene, control cells were transfected with an empty control plasmid. Transfected cells were routinely cultured at 37 °C in DMEM supplemented with 20% FBS, 1% penicillin/streptomycin, 1% L-glutamine, and 300 μg/mL hygromycin. MIO-M1 cells were sub-cultured when they reached about 70% confluency and used between passages 15–20.

Upon verification after one week that the non-transfected control cells did not survive hygromycin exposure, the selection medium was replaced with a maintenance medium containing DMEM with 20% FBS, 1% penicillin/streptomycin, 1% L-glutamine, and 200 μg/mL hygromycin. This adjustment was made to avoid the potential toxicity of prolonged exposure to high hygromycin concentrations, even in stably transfected cells.

### 4.2. Evaluation of the Firefly Luciferase Expression

To assess NF-κB activation, the expression of a Firefly luciferase reporter gene was quantified based on the luminescent signal produced and detected using a luminometer. Prior to the assay, the culture medium was removed, and cells were gently washed with phosphate buffer saline (PBS, Merck Life Science, Milan, Italy) before being lysed using Passive Lysis Buffer (Promega, Madison, WI, USA), following the manufacturer’s guidelines (Technical Bulletin 281, Rev. 8/15). Subsequently, 20 µL of the resulting cell lysate was mixed with 100 µL of luciferase Assay Reagent (Promega, Madison, WI, USA) and incubated at 37 °C to initiate the luminescence reaction. The luciferase content was estimated using a calibration curve generated from known quantities of purified Firefly luciferase standard (QuantiLuM, Promega, Madison, WI, USA)), ranging from 10–15 to 10–19 moles. Final values were normalized to the total protein concentration in each sample.

### 4.3. MIO-M1 Cells Treatment

To induce acute hyperglycemic conditions, cells were serum-starved for 24 h in DMEM containing 0.5% FBS, 50 mU/mL penicillin/streptomycin, 2 mM glutamine, and 1% non-essential amino acid solution and then treated with 75 mM D-glucose dissolved in PBS for 24 h in the same low-serum medium, or with an equal volume of the sole PBS for control group. Sorbinil was dissolved in DMSO (Merck Life Science, Milan, Italy) and, where specified, administered to MIO-M1 cells simultaneously with the serum starvation at a final concentration of 20 μM and a final DMSO percentage of 0.05% (*v*/*v*). The control group received the only vehicle, DMSO, at a final concentration of 0.05% (*v*/*v*).

To mimic inflammatory stress, cells were treated with 0.2 nM TNF-α (Merck Life Science, Milan, Italy) dissolved in human serum albumin (HSA) or with an equal volume of HSA alone for the control group.

At the end of the reported incubation periods, cells were lysed as indicated for future analysis. Total protein concentration was determined by Bradford assay (Bradford, 1976), using a Bio-Rad protein assay kit.

### 4.4. MIO-M1 Cells Viability

To evaluate the cytotoxic effect of acute hyperglycemia on MIO-M1 cells, a modified MTT assay was performed, following the method originally described [23]. Briefly, cells were cultured in 24-well plates at a density of 18,000 cells/cm^2^ and exposed to 75 mM D-glucose for either 24 or 48 h. At each time point, the medium was removed and cells were incubated with 0.5 mg/mL MTT solution (Merck Life Science, Milan, Italy) at 37 °C for 30 min in a humidified 5% CO_2_ atmosphere. After incubation, the formazan crystals were dissolved by the addition of an equal volume of isopropanol containing 0.04 N HCl, and the absorbance at the wavelength of 563 nm was measured through a microplate reader.

### 4.5. Western Blotting

For the Western blot assay, MIO-M1 cell extracts were collected as follows. Phenylmethylsulfonide fluoride (PMSF) was from Merck Life Science (Milan, Italy). All inorganic chemicals were of reagent grade, from VWR (Poole, Dorset, UK) After medium removal, cells were washed with PBS containing protease and phosphatase inhibitors (10 mM NaF, 10 mM Na_4_O_7_P_2_, 2 mM Na_3_VO_4_, 33 mM β-glycerophosphate, and 1 mM PMSF, final concentrations). Then, cells were incubated with Lysis Buffer (Cell Signaling, Danvers, MA, USA) for 5 min at room temperature. The obtained cell lysates were collected and incubated on ice for an additional 10 min, followed by centrifugation at 14,000× *g* for 10 min at 4 °C. A total of 20 µg of protein was mixed (1:1, *v*:*v*) with 4× Laemmli Reducer supplemented with 10% (*w*/*v*) SDS and 0.7 M β-mercaptoethanol, then heated at 70 °C for 10 min and cooled on ice before loading. The protein content was quantified by using Bradford’s assay [24]. Twenty micrograms of protein from each sample were separated by SDS-PAGE (12%) prepared using the TGX Stain-Free™ FastCast™ Acrylamide Kit (Bio-Rad Laboratories, Hercules, CA, USA). Proteins were transferred onto a 0.2 μm PVDF membrane, using the precast Trans-Blot Turbo Transfer Pack Midi kit (Bio-Rad) at 25 V and 1.3 A for 6 min, using a Trans-Blot Turbo Transfer System (Bio-Rad). Membranes were blocked with 5% (*w*/*v*) non-fat dry milk in 50 mM Tris-HCl buffer pH 7.5, containing 150 mM NaCl and 0.1% (*v*/*v*) Tween^®^ 20 (TBS-T) for 1 h at room temperature and subsequently incubated overnight at 4 °C with mouse anti-AKR1B1 (OriGene, Rockville, MD, USA, cat. number: TA346874, diluted 1:1000 in 5% milk/TBS-T) or rabbit anti-COX-2 (Cell Signaling, cat. number: 4842, diluted 1:1000 in 5% milk/TBS-T) primary antibodies. Subsequently, membranes were washed in TBS-T and incubated for 1 h at room temperature with HRP-conjugated secondary anti-mouse antibody (Cell Signaling, cat. number: 7076, diluted 1:1000 in 5% milk/TBS-T) or anti-rabbit (Cell Signaling, cat. 7074, dilution 1:1000 in 5% milk/TBS-T) antibodies.

Blots were developed using the Immobilon™ Western chemiluminescent HRP substrate (Bio-Rad), and signals were detected with a CCD-based ChemiDoc System device (Bio-Rad). The optical density (OD) of the target bands was evaluated by ImageLab 3.0 software (Bio-Rad). The data were normalized to the corresponding OD of total protein content visualized via stain-free technology (Bio-Rad).

### 4.6. Enzymatic Determination

For the enzymatic assay, MIO-M1 cells were rinsed with PBS containing 1 mM PMSF, then collected using a scraper and transferred to an Eppendorf tube. Cellular lysis was performed by three rapid F/T cycles, followed by centrifugation at 4 °C for 15 min at 14,000× *g*. The supernatant was retained, and protein content was quantified for the subsequent normalization. AKR1B1 activity was measured following previously established protocols [25], by measuring the decrease in absorbance at 340 nm due to NADPH oxidation (ε_340_ = 6.22 mM^−1^·cm^−1^) at 37 °C using a Biochrom Libra S32 spectrophotometer (Biochrom Ltd., Cambridge, UK). The standard reaction mixture (final volume of 0.7 mL) included 0.25 M of sodium phosphate buffer, a pH of 6.8, 0.5 mM of EDTA, 0.38 M of ammonium sulfate, 0.18 mM of NADPH, and 10 mM of L-idose as a specific substrate.

SORD activity was measured as described by Sardelli et al. [13], by monitoring the decrease in absorbance at 340 nm due to NADH oxidation (ε_340_ = 6.22 mM^−1^·cm^−1^) at 37 °C using a Biochrom Libra S32 spectrophotometer. The standard reaction mixture (final volume of 0.7 mL) included 100 mM of Tris/HCl buffer at a pH of 7.4, 0.24 mM of NADH, and 0.4 M of fructose as a substrate. The enzymatic reaction was initiated by the addition of the substrate. One unit of enzymatic activity was defined as the amount of enzyme required to convert 1 μmol of substrate per minute under these specific assay conditions.

### 4.7. Determination of Sorbitol Content

To quantify the intracellular sorbitol content, an in-house spectrofluorimetric assay using purified SORD as an ancillary enzyme was employed. Firstly, MIO-M1 cells were rinsed with PBS containing 1 mM PMSF, then collected using a scraper and transferred to an Eppendorf tube. Cellular lysis was performed by three rapid F/T cycles, followed by centrifugation at 4 °C for 15 min at 14,000× *g*. The supernatant was retained, and protein content was quantified for the subsequent normalization. Samples underwent protein precipitation with the addition of 1 M perchloric acid and centrifugation at 4 °C for 5 min at 14,000× *g*. The resulting supernatant was brought to pH 7 by adding KOH, and again centrifuged at 4 °C for 15 min at 14,000× *g*. For the determination of sorbitol content, aliquots of the prepared supernatant were added to a reaction mixture (final volume 200 μL) containing 0.24 mM NAD+ and 150 mU of purified SDH in 100 mM Tris-HCl buffer, pH 8. The reaction was initiated by the addition of NAD+. Fluorescence was measured at an emission wavelength of 460 nm, with excitation at 355 nm, for a total time of 10 min at 37 °C. Sorbitol concentration was determined using a calibration curve obtained by performing the reaction described with known D-sorbitol concentrations ranging from 5 to 40 µM, and the results were normalized to the protein content of the samples.

### 4.8. Statistical Analysis

Data are presented as mean ± SEM of independent experiments. Comparisons were made using Student’s *t*-test or by ANOVA when appropriate, followed by Tukey’s Multiple Comparison post hoc test or Dunnett’s test to compare the difference between two groups. Values of *p* ≤ 0.05 were considered statistically significant. GraphPad Prism Software, version 6.07 (GraphPad Software, Inc., La Jolla, CA, USA), was used to perform statistical analysis.

## Data Availability

The original contributions presented in this study are included in the article and Supplementary Material. Further inquiries can be directed to the corresponding author.

## Supplementary Materials

The following supporting information can be downloaded at: https://www.mdpi.com/article/10.3390/ijms26146741/s1.

## Abbreviations

The following abbreviations are used in this manuscript:

MGCs	Müller glial cells
AKR1B1	Aldose reductase
DR	Diabetic retinopathy
SORD	Sorbitol dehydrogenase
MTT	3-(4,5-dimethylthiazol-2-yl)-2,5-diphenyltetrazolium bromide
