# Supplementary material for: Acute Hyperglycemia-Induced Inflammation in MIO-M1 Cells: The Role of Aldose Reductase
Source: Int J Mol Sci. 2025 Jul 14;26(14):6741. doi: 10.3390/ijms26146741 (PMC12295778; doi:10.3390/ijms26146741)
Supplement: Supplementary file 1 [file ijms-26-06741-s001.zip › ijms-3742030-supplementary.pdf]

Acute Hyperglycemia-Induced Inflammation in MIO-M1 cells: the role of Aldose Reductase

Francesca Felice, Gemma Sardelli, Francesco Balestri, Lucia Piazza, Mario Cappiello, Antonella Del Corso, Simone Allegrini and Roberta Moschini

SUPPLEMENTARY MATERIALS

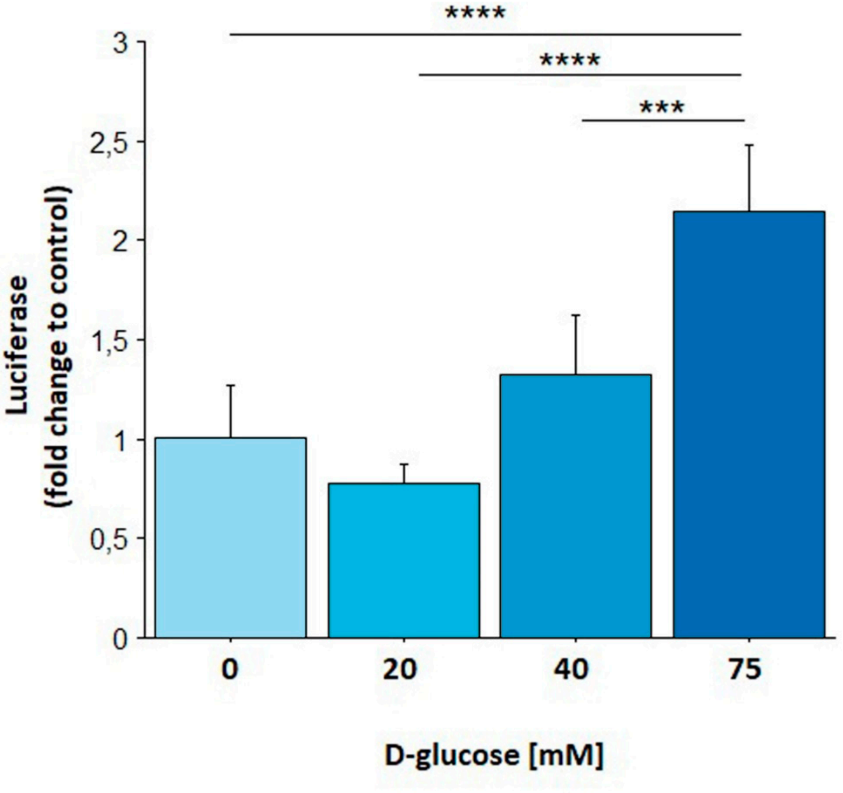

Figure 1S. Effect of glucose on NF-κB activation in MIO-M1 cells.

MIO-M1 cells were transfected with an NF-κB luciferase reporter and treated with increasing concentrations of D-glucose (0, 20, 40, and 75 mM) for 24 h. NF-κB activity was measured via luciferase assay and expressed as fold change relative to the control condition (0 mM glucose). A significant increase in NF-κB activity was observed only at 75 mM glucose. Data represent mean ± SEM of n = 5 independent experiments. Statistical analysis was performed through one-way ANOVA followed by Dunnett post hoc test (***$p < 0.001$, ****$p < 0.0001$).

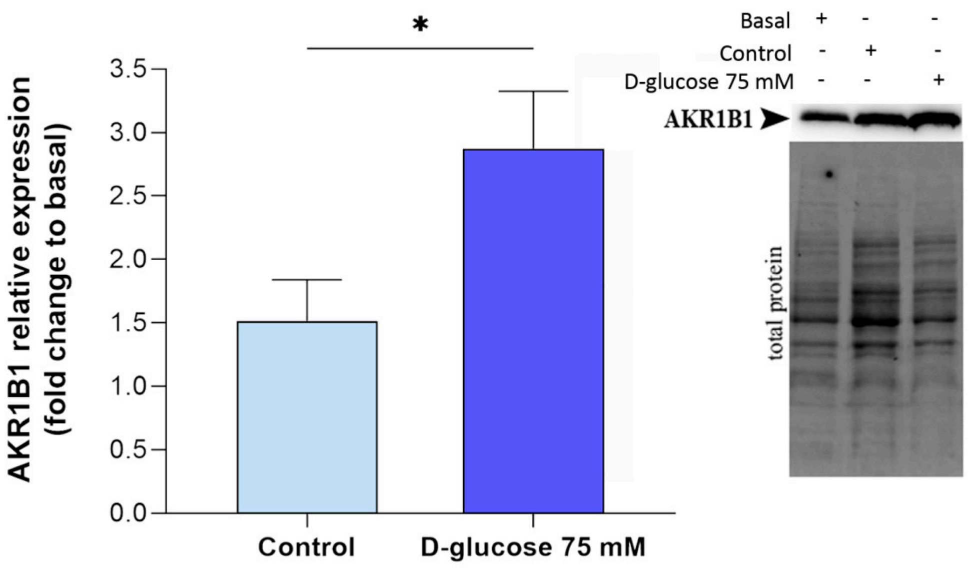

Figure 2S. Effect of acute hyperglycemia on AKR1B1 protein expression. MIO-M1 cells were incubated with or without 75 mM D-glucose for 24 hours. Cells were harvested and AKR1B1 protein expression was determined through Western Blot analysis. Densitometric analysis of AKR1B1 expression, normalized to total protein, was reported as fold-change of AKR1B1 band expression to basal expression condition. Data are reported as mean ± SEM of at least three independent experiments. Statistical analysis was performed through Student's T-test (* $p \leq 0.05$ vs control).
